# Are Existing Monocular Computer Vision-Based 3D Motion Capture Approaches Ready for Deployment? A Methodological Study on the Example of Alpine Skiing

**DOI:** 10.3390/s19194323

**Published:** 2019-10-06

**Authors:** Mirela Ostrek, Helge Rhodin, Pascal Fua, Erich Müller, Jörg Spörri

**Affiliations:** 1 Computer Vision Laboratory, École Polytechnique Fédérale de Lausanne, 1015 Lausanne, Switzerland; mirela.ostrek@gmail.com (M.O.); helge.rhodin@ubc.ca (H.R.); pascal.fua@epfl.ch (P.F.); 2 Faculty of Electrical Engineering and Computing, University of Zagreb, 10000 Zagreb, Croatia; 3 Department of Computer Science, University of British Columbia, Vancouver, BC V6T 1Z4, Canada; 4 Department of Sport Science and Kinesiology, University of Salzburg, 5400 Hallein-Rif, Austria; erich.mueller@sbg.ac.at; 5 Department of Orthopedics, Balgrist University Hospital, University of Zurich, 8008 Zurich, Switzerland

**Keywords:** biomechanics, human pose estimation, markerless tracking, video-based 3D kinematics, technical validation, alpine ski racing

## Abstract

In this study, we compared a monocular computer vision (MCV)-based approach with the golden standard for collecting kinematic data on ski tracks (i.e., video-based stereophotogrammetry) and assessed its deployment readiness for answering applied research questions in the context of alpine skiing. The investigated MCV-based approach predicted the three-dimensional human pose and ski orientation based on the image data from a single camera. The data set used for training and testing the underlying deep nets originated from a field experiment with six competitive alpine skiers. The normalized mean per joint position error of the MVC-based approach was found to be 0.08 ± 0.01 m. Knee flexion showed an accuracy and precision (in parenthesis) of 0.4 ± 7.1° (7.2 ± 1.5°) for the outside leg, and −0.2 ± 5.0° (6.7 ± 1.1°) for the inside leg. For hip flexion, the corresponding values were −0.4 ± 6.1° (4.4° ± 1.5°) and −0.7 ± 4.7° (3.7 ± 1.0°), respectively. The accuracy and precision of skiing-related metrics were revealed to be 0.03 ± 0.01 m (0.01 ± 0.00 m) for relative center of mass position, −0.1 ± 3.8° (3.4 ± 0.9) for lean angle, 0.01 ± 0.03 m (0.02 ± 0.01 m) for center of mass to outside ankle distance, 0.01 ± 0.05 m (0.03 ± 0.01 m) for fore/aft position, and 0.00 ± 0.01 m^2^ (0.01 ± 0.00 m^2^) for drag area. Such magnitudes can be considered acceptable for detecting relevant differences in the context of alpine skiing.

## 1. Introduction

In the last decade, technological evolutions have significantly impacted various fields of science and daily life. This is particularly true for the area of sports, where recent advances in measurement technology have enabled the extraction of a variety of novel performance-, load-, and health-related metrics. However, despite these technological advances, collecting accurate and precise three-dimensional (3D) kinematic data under real-life conditions remains challenging, since for many outdoor sports, such as alpine skiing, field experiments and large capture volumes are crucial for obtaining valid results with practical and/or clinical relevance.

Since the feasibility of collecting 3D kinematic data on a ski track by the use of marker-based optoelectronic stereophotogrammetry (golden standard under laboratory conditions) has been demonstrated to be strongly limited [1], recent biomechanical studies in alpine skiing have primarily incorporated systems with multiple panned/tilted/zoomed video cameras (i.e., video-based stereophotogrammetry) and/or wearable measurement technologies [2,3,4,5,6,7,8,9,10,11,12,13,14,15,16,17,18,19,20]. The major advantage of video-based stereophotogrammetry is its superior position determination accuracy and precision in connection with 3D human pose estimation [2]; however, their disadvantages are the complex multi-camera setup and extensive post processing (i.e., camera calibration and manual annotation) demands, and, compared to wearable measurement technologies, their limited capture volumes.

One potential solution to overcome the limitation of requiring complex camera calibration procedures and extensive manual annotation efforts when using video-based stereophotogrammetry for in-field 3D human pose estimation might be found in a deep learning approach, as was proposed most recently in Rhodin et al. [21]. This becomes feasible when there is a sufficiently large and representative manually annotated dataset to properly train the underlying algorithms. The basic idea behind this is to use existing video-based stereophotogrammetric datasets (i.e., manually annotated and calibrated multiple cameras views from different perspectives) for the training of a deep network. Subsequently, this network is used to predict the true 3D pose based on the two-dimensional (2D) images of a single uncalibrated camera under similar assessment setups.

It goes without saying that once such a monocular computer vision (MCV)-based approach is able to derive relevant metrics with sufficiently high accuracy and precision, conducting 3D biomechanical field experiments under real-life conditions (e.g., collecting kinematic data on a ski track) could be revolutionized. However, it is not a priori clear whether the high standards of 3D position determination accuracy and precision demonstrated for conventional approaches can also be achieved by automatized annotation and trained deep nets. Moreover, for a conclusive discussion of the deployment readiness of MCV for application-orientated research, knowledge about the accuracy and precision of the estimated joint angles and application-specific metrics would be key and has not been thoroughly analyzed yet. Existing approaches, such as that of Rhodin et al. [21], primarily focus on human pose estimation, but neglect the sports equipment and relevant skiing-specific metrics, such as lean angle, fore/aft position, and air drag. Furthermore, algorithm training and testing rely on a weakly-supervised machine learning method and a very small data set only.

Accordingly, the aims of this study are threefold: (1) to thoroughly investigate the 3D joint position determination errors of a fully supervised MCV-based approach (automated annotation and single-view cameras) in comparison to the golden standard reference method of video-based stereophotogrammetry (manual annotation and calibrated multi-view cameras) and to set a thorough baseline for future advances in the field; (2) to implement and assess the accuracy and precision of skiing-specific metrics that, to the best of our knowledge, have not yet been analyzed in the context of deep learning and MCV; and (3) to explore the practical usability and deployment readiness of the investigated MCV-based approach for alpine skiing-related biomechanical field experiments with reference to existing solutions.

## 2. Materials and Methods

### 2.1. Measurement Protocol and Experimental Setup

**Figure 1:**
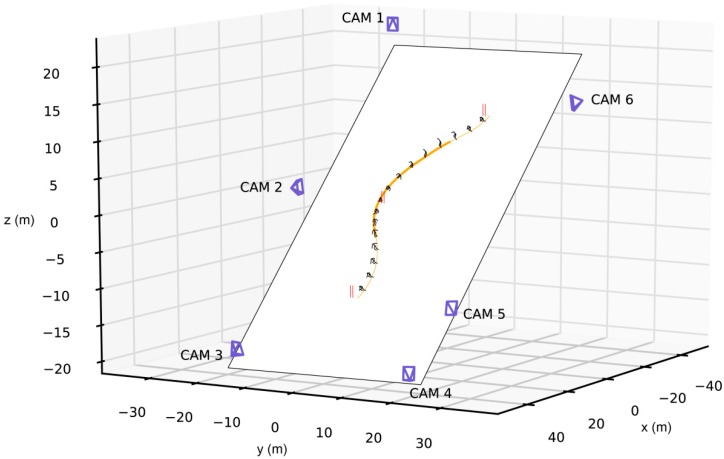
Setup overview visualizing the camera and gate positions measured in meters, as well as the capture volume spanned by three consecutive giant slalom gates. The small person size in relation to the camera distance highlights the difficulty in estimating skiing poses accurately.

In the framework of a biomechanical field experiment, six elite competitive alpine skiers performed four runs on a typical giant-slalom (GS) course (average gate distance: 27 m; average gate offset: 8 m; slope inclination angle: 26°; water-injected snow surface). In the middle of the course, a complex multi video-camera set-up covered a capture volume spanned by three consecutive GS gates (Figure 1). The capture volume was surrounded by a total of 142 reference points with geodetically measured 3D positions (Leica Total Station 1200). The study protocol was approved by the ethics committee of the Department of Sport Science and Kinesiology at the University of Salzburg.

### 2.2. Data Collection and Processing (Reference Method)

**Figure 2:**
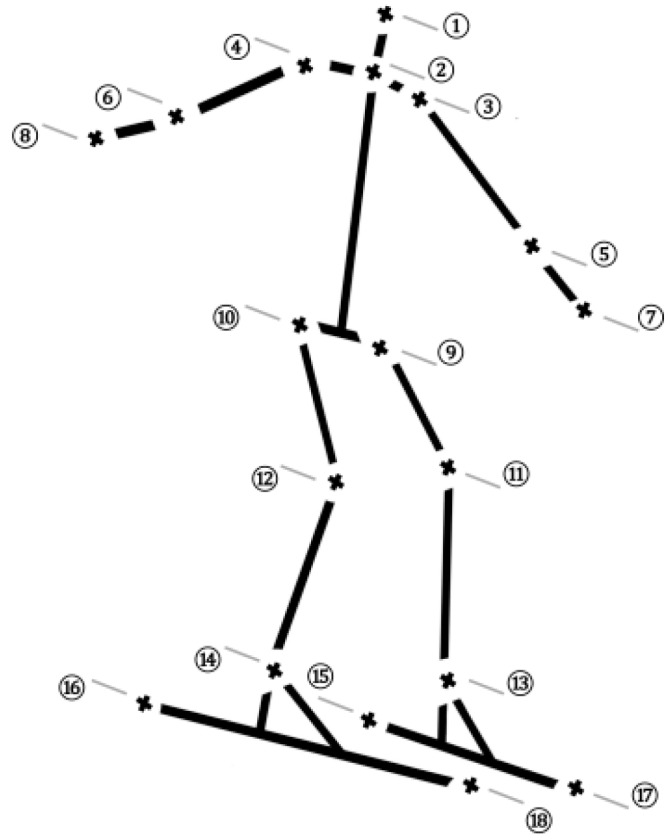
Segment model, including all 18 joint centers and connecting segments considered for analysis. 1: head; 2: neck; 3: left shoulder; 4: right shoulder; 5: left elbow; 6: right elbow; 7: left hand; 8: right hand; 9: left hip; 10: right hip; 11: left knee; 12: right knee; 13: left ankle; 14: right ankle; 15: left ski tail; 16: right ski tail; 17: left ski tip; 18: right ski tip.

Within the capture volume (i.e., the section spanned by three consecutive GS gates in the middle of the course) the skiers were simultaneously filmed by six panned tilted and zoomed high-definition video-cameras (Sony PMW-EX3, 50Hz, gen-lock synchronized, Tokyo, Japan). In each frame of each camera, an 18-point body segment model (Figure 2) and skier-ambient reference points were manually annotated by an experienced evaluator in custom-made software (Volker Drenk, Leipzig Germany). Corresponding annotations and known 3D positions of the reference points and cameras were then used to reconstruct the body segment model in 3D. For the determination of the instant direct linear transformation (DLT) constants, a panning algorithm was applied [22]. If, despite the various camera perspectives, a 3D reconstruction of certain landmarks of the segment model was not feasible, gaps were interpolated using a NEXUS (Vicon Motion Systems Ltd, Oxford, UK) implemented pattern fill, which builds upon ambient, visible, and rigidly-connected landmarks. Finally, all data were filtered by the use of a second-order Butterworth low-pass filter with cut-off frequencies determined according to the Jackson knee method [23], and body segments were normalized by the use of an iterative segment length normalization routine proposed by Smith [24]. This reference method can be considered the golden standard for collecting kinematic data on ski tracks and has been shown to be both valid and reliable. In a previous study and for a comparable setup on a ski track, the photogrammetric errors were reported to be ~2 cm or less [2].

### 2.3. Monocular Computer Vision-Based 3D Human Pose Estimation Algorithm

Our MCV-based approach was based on the most recent advances in artificial intelligence and deep learning for predicting a person’s 3D pose directly from 2D images [25]. All of these methods optimize the parameters of a neural network using a large dataset of example input images with manually annotated and reconstructed 3D pose labels. Once optimized, the neural network parameters are kept fixed, no manual 3D pose annotation is required at test time. Given a new test image as input, the neural network directly computes the sought 3D pose by generalizing from the examples seen during training.

In the current study, we deployed the recently proposed MCV method by Rhodin et al. [21], which applies deep learning for pose estimation in alpine skiing. It took images of resolution 256 × 256 px as input, used the 50-layer version of the residual network architecture of He et al. [25] for processing, and output the subject’s 3D joint location relative to the pelvis. Subsequently, it computed ski metrics by introducing the corresponding camera-relative calculus, which was one of the new contributions of the current study. In a preliminary study, we tried to predict biomechanical variables directly from the image, without inferring 3D pose, however, with insignificant improvement in accuracy.

The ResNet50 started with a 7 × 7 convolutional filter and subsequent 3 × 3 max pooling. Subsequently, we used four bottleneck-residual blocks, each consisting of three, four, six, and three layers. A layer applied a 1 × 1 convolution to compress to the bottleneck resolution, a 3 × 3 convolution at that resolution, and a 1 × 1 convolution to expand the number of channels four-fold. The bottleneck resolution of the four blocks was, respectively, 64, 128, 256, and 512 channels, and the layer 3 × 3 convolution stride was 1, 2, 2, and 2. Finally, the network output was predicted with a fully-connected layer that took the output of the last residual block followed by the global average pooling layer as input. The first three residual blocks of layers were initialized through transfer learning by pre-training the model on a 2D pose estimation task, as proposed by Mehta et al. [26], and were kept constant for the ski-specific training. The network was trained using the Adam optimizer with a constant learning rate of 0.001, a least squares loss, and early stopping. The early stopping was necessary, since the large capacity residual network over fitted on the medium sized ski dataset when trained for too many iterations, potentially leading to less accurate reconstructions. Moreover, the Adam optimization method is stochastic and the accuracy fluctuates, which requires the ideal stopping point for each training run to be determined precisely and individually. While the network architecture and optimization method were equivalent to Rhodin et al. [21], the current methodology primarily differed in the following points: (1) the model was trained from scratch; (2) the output layer and dataset annotation were extended to output the 3D location of the skis on top of the subject joint locations; (3) end-to-end training was facilitated with a single loss function instead of multiple self-supervised objectives; (4) the training pose distribution was whitened (mean subtraction and standard deviation normalization) instead of predicting a scale-normalized pose; and (5) a larger training set was used, and a 6-fold cross validation setup with clear training, validation, and test sets. Thereby, the early stopping hyperparameter was determined on subjects that were strictly separated from the test set while we were still evaluating on all available runs. This improved training. The testing procedure is explained in more detail in the following section.

### 2.4. Algorithm Training and Testing

For the purposes of the algorithm training and testing the data, all 24 captured trials were divided into training, validation, and test sets, as outlined in Table 1. A trial refers to one run across the analyzed capture volume spanned by three consecutive GS gates. Trials of the same subject (S1–S6) were incorporated into the same set, to ensure strict separation between training, validation, and testing. The training set was used to train the parameters of the employed neural network. The validation set was needed to select free hyper parameters, in our case the number of training iterations. The accuracy and precision were evaluated on the separate test set, i.e., the trials of a subject not considered for training or validation.

The algorithm training and validation were based on the entire capture volume spanned by three consecutive GS gates and all six cameras. For testing, only one turn cycle (i.e., the data from the turn-switch between the first and second gate to the next turn-switch between the second and third gate, see Figure 1) was used and we experimented with subsets of cameras. We also tried using all available frames, but with insignificant differences. Table 1 lists the exact number of training, validation, and test samples. The major advantage of such a procedure (i.e., using a single turn for testing) is the unique opportunity of being able to directly compare the accuracy and precision of wearable measurement technologies assessed in previous studies [16,17,19,20] with those obtained with the MVC-based approach in this study, as the underlying reference data set was then equivalent. Six-fold cross validation was used to report results on all available sequences while maintaining this strict separation. Each split (Split 1–6) defined unique training, validation, and test sets, as shown in Table 1. One deep learning model was trained for each of them, and the reported results were aggregated from all splits.

Compared to Rhodin et al. [21], the current study is more detailed and more conclusive. The original had no separation of validation and test sets, reported results only on a single validation subject, and used a substantially smaller training dataset (i.e., 12 GS sections; two per subject) than the current one. Moreover, in this study, additional relevant skiing-specific metrics were implemented and tested, as defined below.

### 2.5. Calculation of Selected Metrics

The MCV algorithm output the $N_{J}=18$ key-point locations (i.e., the major human joints and the ski tips and tails, Figure 2) visible in the image, represented as a single pose matrix $P\;\in \;R^{3\times N_{J}}$. This is a different approach to the method of Rhodin et al. [21], which did not output the ski key-points needed to formulate the ski-specific metrics that require a local coordinate system relative to the ski. All subsequently described metrics were computed as a function of these predicted position variables.

### 2.6. 3D Joint Position Determination Error

Mean per joint position error (MPJPE) was calculated as the average differences in 3D joint positions that were reconstructed by the MVC-based approach and the reference method. Because monocular reconstruction is inherently scale ambiguous, we followed previous work and reported the normalized metric (NMPJPE). It is defined as the MPJPE after normalizing the predicted positions in scale to the reference (see Rhodin et al. [21]).

### 2.7. Knee and Hip Flexion Angle

Knee flexion angle (*θ_Knee_*) and hip flexion angle (*θ_Hip_*) were determined based on the given 3D human pose *P*. The *J*th column of *P* denotes the position of key-point *J* relative to the pelvis and further will be referred to the 3D position of joint *J* as *P_J_*. For the turn, outside leg knee flexion angle (*θ_Knee,outside_*) was defined as follows:

(1) $$v_{1}\;=\;P_{Hip,\;outside}\;-\;P_{Knee,\;outside};$$

(2) $$v_{2}\;=\;P_{Ankle,\;outside}\;-\;P_{Knee,\;outside};$$

(3) $$\theta_{Knee,\;outside}=arccos\frac{v_{1}\;\cdot \;v_{2}}{\left\|v_{1}\|\|v_{2}\right\|}.$$

Hip flexion angle for the turn outside leg (*θ_Hip, outside_*) was calculated similarly, whereas:

(4) $$v_{1}\;=\;P_{Neck}\;-\;\frac{P_{Hip,\;outside}\;-\;P_{Hip,\;inside}}{2};$$

(5) $$v_{2}\;=\;P_{Knee,outside}\;-\;P_{Hip,outside}.$$

In an equivalent way, the same angles for the turn inside leg were calculated (*θ_Knee,inside_; θ_Hip,inside_*).

### 2.8. Skiing-Specific Metrics

For the assessment of the feasibility of incorporating MCV for application-orientated research in the context of alpine skiing, selected skiing-specific metrics were calculated. In earlier studies, such metrics were typically used for performance- and injury-related assessments [3,11,14,27].

Relative center of mass (COM) position *(p_COM,rel_)* was calculated as the sum of the masses *m_i_* of the individual body segments multiplied by the corresponding pose vectors *P_i_* and divided by the total mass of the human body M:

(6) $$p_{COM,rel}\;=\;\frac{1}{M}\;\sum_{i=1}^{N_{J}}m_{i}P_{i},$$

where the average body weight distribution has been taken from Clauser, et al. [28].

**Figure 3:**
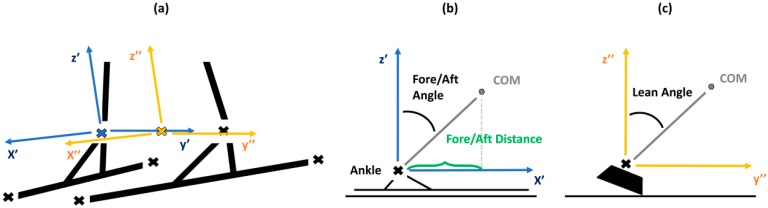
Definitions of the fore/aft position (*d_Fore/Aft_*) and the lean angle (*λ_Lean_*). (**a**) Local coordinate systems are defined relative to the ski and up direction. (**b**) The fore/aft position is the projection of the COM on the x’ axis. (**c**) The lean angle is the orientation of COM in the x”-y” plane.

For the calculation of the COM to outside ankle joint center distance *(d_vertical_)* and fore/aft position *(d_Fore/Aft_),* a local coordinate system (x’, y’, z’) was used. Previous studies set the origin of this coordinate system into the ankle joint of the outside leg, whereas x’ was defined by the direction of the longitudinal axis of the ski, z’ was perpendicular to the slope plane, and y’ resulted from forming a right-handed triad with x’ and z’ (Figure 3) [11,14]. In our monocular case, the global camera location in relation to the slope was unknown and we targeted full automation. Therefore, we determined the outside ski automatically based on whether the COM was closer to the left or right leg. This strategy proved to be reliable. In our test sequences, it coincided with the manual annotations of crossing points, with zero to two frames differences only. Moreover, the up-vector z’ was computed as the vector that was perpendicular to the ski axis and left to right ankle direction. This strategy assumes that the skis were perpendicular to the ground, which is even approximately true during jumps and necessary because the slope direction is unknown for uncalibrated pan-tilt-zoom cameras. Subsequently, y’ was computed as before to form a right-handed coordinate system.

Having derived the inside leg, as well as the local coordinate system based on the ski orientation, ski-specific metrics were computed as suggested in previous studies (Figure 3): *d_vertical_* was calculated as the length of the vector connecting the ankle with COM; *d_Fore/Aft_* was defined as the sine of the fore/aft angle, which is the angle between the z’-axis and the ski-COM vector projected to the x’−z’ plane [3,11,14]. For determining the skier’s lean angle *(λ_Lean_)*, a local coordinate system at the mid ankle was incorporated (x’’, y’’, z’’). *λ_Lean_* was then computed as the angle between the z’’-axis and the ski-COM vector projected to the y’’−z’’ plane (Figure 3c), as done previously [3,11].

The drag area *(C_D_A)_BARELLE_* was calculated based on the reconstructed 3D human posture of the skiers in accordance with the experimental model of Barelle, et al. [29]:

(7) $${\left(C_{D}A\right)}_{BARELLE}\;=\;0.003\cdot h-0.026+0.041\cdot 2w,$$

where *h* is the ankle to shoulder distance projected on the upwards vector that is orthogonal to the ground, and *w* the arm span projected on the plane orthogonal to the velocity vector and normalized by the total arm span. These computations are equivalent to the angle-based height and width measures computed from five angles and three segment lengths as described in Barelle, Ruby, and Tavernier [29]. We introduced the vector–based formulation as it is easier to compute from joint location estimates. Note that these computations go far beyond simple joint position and angle measurements evaluated by Rhodin et al. [21], as those do not require computation of ski key-points and corresponding local, ski-relative coordinate system nor do they depend on knowledge of the inside or outside ski.

### 2.9. Statistical Analysis

**Figure 4:**
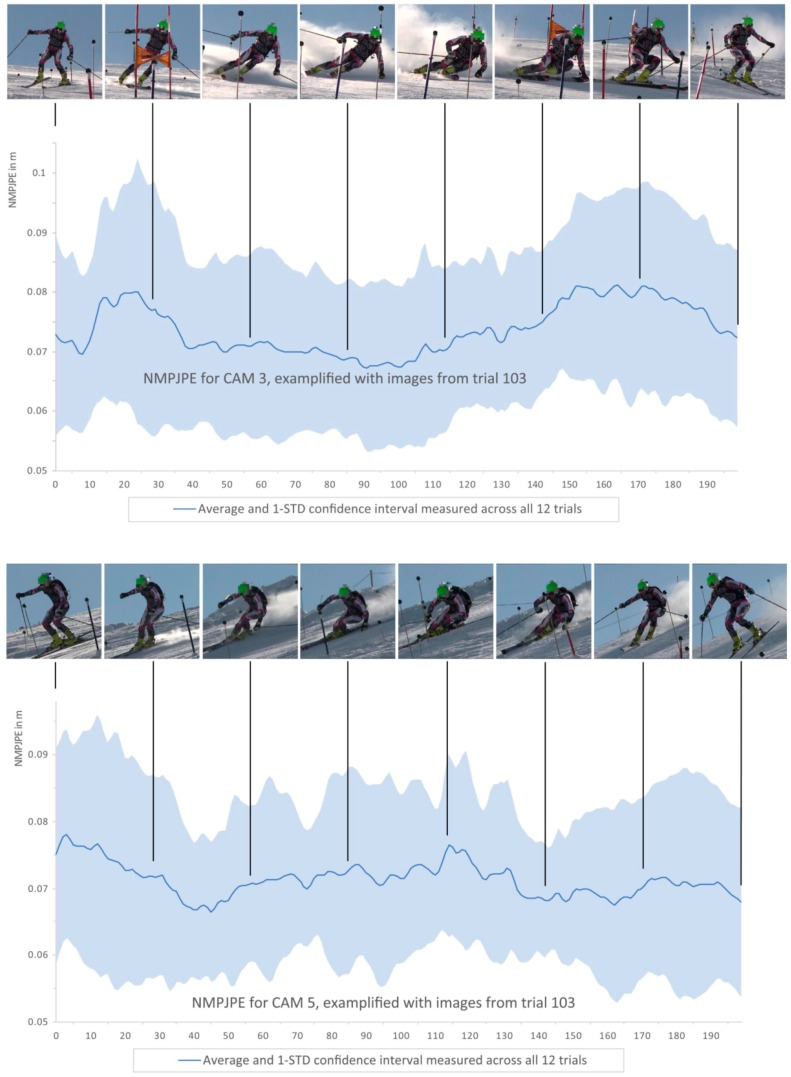
Normalized mean per joint position error (NMPJPE) for the best camera perspectives (CAM 3 and CAM 5; frontal-lateral) over one turn cycle.

NMPJPE was reported as mean ± standard deviation (SD) and was plotted over one entire giant slalom turn cycle (from one turn-switch to the next turn-switch) for the cameras with the lowest average errors, i.e., CAM 3 and CAM 5 (Figure 4). In order to analyze the mean error and the standard deviation (SD) across different trials in relation to characteristic turn phases, underlying data were time normalized through re-sampling and linear interpolation. The resulting 200 normalized points were used to compute the mean and SD values independently across each of the 200 time points. For assessing the systematic and random errors when estimating the knee/hip flexion angles and other skiing-specific metrics, accuracy was defined as the arithmetic mean of the differences between the same metrics obtained by the MCV-based approach and the golden standard reference method. Precision was calculated as the standard deviation of the accuracy observed across all 24 trials tested during cross validation, as defined in Fasel, et al. [16]. For the vector-valued *p_COM,rel_*, the mean Euclidean distance to the reference was used as the accuracy measure.

## 3. Results

### 3.1. 3D Joint Position Determination Error

In Table 2, an overview of the NMPJPE for an MCV-based 3D reconstruction using different camera perspectives is given. The data show the individual cross-validation results, split individually for S1–6, and the overall mean and SD.

For single camera 3D joint position estimation, on average (i.e., over all cameras), NMPJPE was found to be 0.08 ± 0.01 m, whereas for some cameras slightly better values were observed (CAM 3: 0.07 ± 0.01 m, CAM 5: 0.07 ± 0.00 m). Looking at the time-error progression of those cameras (Figure 4), NMPJPE remained more or less constant over the entire analyzed turn cycle. However, one can also recognize that the two peak values in mean NMPJPE correspond to those phases in which the panned and tilted camera views approximated a pure frontal or lateral perspective of the skier, as shown by the input images associated to the different phases of the turn (Figure 4). The largest NMPJPE values were observed for CAM 1, which was filming the skier entirely from behind. Most accurate were CAM 3 and CAM 5, with an NMPJPE of 0.07 ± 0.02 m. The observed standard deviation was relatively low, scaling consistently to roughly one third of the measured absolute error.

### 3.2. Knee and Hip Flexion Angle

The accuracy and precision values for the estimation of knee and hip flexion angles based on the monocular 3D reconstruction are presented in Table 3. For knee flexion angles, accuracy and precision (in parenthesis) were revealed to be 0.4 ± 7.1° (7.2 ± 1.5°) for the turn outside leg, and −0.2 ± 5.0° (6.7 ± 1.1°) for the turn inside leg. For hip flexion angles, the corresponding values were −0.4 ± 6.1° (4.4° ± 1.5°) and −0.7 ± 4.7° (3.7 ± 1.0°), respectively. These values were computed over all 24 trials viewed from CAM 3 and CAM 5, as these were determined as the optimal camera positions in the preceding joint position error experiment. The mean errors observed across the test subjects were consistent, with one exception: subject 4 showed relatively high mean errors in the knee flexion angle of the inside leg.

### 3.3. Skiing-Specific Metrics

As illustrated in Table 4, the accuracy and precision (in parenthesis) of skiing-related metrics was found to be 0.03 ± 0.01 m (0.01 ± 0.00 m) for *p_COM,rel_*, −0.1 ± 3.8° (3.4 ± 0.9) for *λ_Lean_*, 0.01 ± 0.03 m (0.02 ± 0.01 m) for *d_vertical_*, 0.01 ± 0.05 m (0.03 ± 0.01 m) for *d_Fore/Aft_*, and 0.00 ± 0.01 m^2^ (0.01 ± 0.00 m^2^) for *CDA_BARELLE_*. Here, no outlier was visible for subject 4.

## 4. Discussion

### 4.1. 3D Joint Position Determination Errors of the Monocular Computer Vision Approach

Compared to the in-field golden standard method of video-based stereophotogrammetry (manual annotation and multi-view cameras), the NMPJPE of the proposed MCV-based approach (fully-automated annotation and single-view cameras) was found to be 0.08 ± 0.01 m (Table 2). Thus, the determination of 3D joint positions by the use of MCV may suffer from errors, which are around the same or lower than the magnitude reported for wearable measurement technologies in the context of alpine skiing: on average, absolute joint position accuracy (0.13 m) and precision (0.07 m) for inertial measurement unit (IMU) and global navigation satellite system (GNSS) fusion [16], and relative joint position accuracy (0.08 ) and precision (0.02 m) for an IMU-based estimation [30]. The same applies to the accuracy and precision of the resulting relative center of mass position *(p_COM,rel_),* 0.03 m and 0.01 m, also approximately the same or lower order of magnitude as observed for wearable measurement technologies, i.e., 0.08 m and 0.06 m (model without arms) [16], and 0.03 m and 0.01 m (model with arms) [30]. In particular, the findings of Fasel et al. [16] are highly comparable with those of the current study (Table 4), since exactly the same reference method and measurement setup was used.

The lowest NMPJPEs were found for CAM 3 (0.07 ± 0.01 m) and CAM 5 (0.07 ± 0.00 m). For the current setup, these two cameras may be considered the best perspectives for reducing the occurring 3D joint position determination errors. Both had in common that they were always filming the skiers sideways from the front; however, with minimal areas where the panned and tilted cameras had a full frontal or full lateral perspective on the skiers’ anatomical frontal plane (see the header of Figure 4). Moreover, it is interesting to observe that for these two cameras, NMPJPE peaks occurred at time points where the panned and tilted camera perspectives had approximately pure frontal or lateral views. In consequence, for the purpose of an MCV-based motion capture of a skier, a frontal, however, slightly lateral (i.e., out of the slope’s fall line) positioned camera might be the most appropriate perspective.

### 4.2. Accuracy and Precision of Estimating Joint Angles by the Monocular Computer Vision Approach

By using the MCV-based approach, knee flexion angles were estimated with an accuracy and precision of 0.4 ± 7.1° (7.2 ± 1.5°) for the turn outside leg, and −0.2 ± 5.0° (6.7 ± 1.1°) for the turn inside leg. For hip flexion angles, the corresponding values were −0.4 ± 6.1° (4.4 ± 1.5°) and −0.7 ± 4.7° (3.7 ± 1.0°), respectively (Table 3). For the turn outside leg and both knee and hip flexion angles, this is comparable to the accuracy and precision observed for drift-reduced inertial sensor-based approaches in previous studies: knee flexion, accuracy 0.1° to 1.7° and precision 3.4° to 4.8°; hip flexion, accuracy 3.8° to 10.7°, precision 3.6° to 6.0° [20,31]. However, the MCV-based systematic error magnitudes on the turn inside leg were only half those observed in Fasel et al. [20] for alpine skiing. This might be explained either by the prominent soft tissue artefacts when using wearable sensors at higher knee flexion angles, as they are present on the turn inside leg while skiing [20], or a potentially higher intra- and inter-trial consistency of the typical motion patterns on the inside leg while skiing and, therefore, higher predictability by trained deep nets. Again, the joint angle related findings of Fasel et al. [20] for inertial sensor-based systems are highly comparable with those of the current study, since the exact same reference method and measurement setup were used.

### 4.3. Are Existing Monocular Computer Vision-Based 3D Motion Capture Approaches Ready for Deployment in the Context of Alpine Skiing?

For skiing-related metrics, the accuracy and precision values were 0.03 ± 0.01 m (0.01 ± 0.00 m) for *p_COM,rel_*, −0.1 ± 3.8° (3.4 ± 0.9) for *λ_Lean_*, 0.01 ± 0.03 m (0.02 ± 0.01 m) for *d_vertical_*, 0.01 ± 0.05 m (0.03 ± 0.01 m) for *d_Fore/Aft_*, and 0.00 ± 0.01 m^2^ (0.01 ± 0.00 m^2^) for *CDA_BARELLE_* (Table 4). Like for the joint angle estimation accuracy and precision values discussed above, such systematic and random error magnitudes can be considered sufficiently low for detecting practically/clinically relevant differences in the context of many skiing performance- and injury-related research questions. In both recreational and competitive alpine skiing, turn average knee flexion angle differences of 5–15° have been reported to be meaningful when comparing different turn techniques or competition disciplines [27,32]. When comparing different course settings in giant slalom and slalom for instance, *λ_Lean_* differences of 1–5°, *d_vertical_* differences of 0.02–0.10 m, and *d_Fore/Aft_* differences of 0.02–0.05 m were found to be practically/clinically relevant [3,11]. Moreover, from wind tunnel tests it is known that even very small differences in the skiing position increase the drag area by up 10% [9]. In view of this, the *CDA_BARELLE_*-related accuracy and precision values observed for the MCV approach in this study, i.e., 0.00 ± 0.01 m^2^ (0.01 ± 0.00 m^2^), appear to be comparably small, knowing that the total drag area in a tucked position is around 0.20 m^2^.

Finally, we would like to stress the superior practicability of the proposed MCV approach we experienced in the context of alpine skiing related experiments, since annotation was automated and after the training of the underlying deep nets only one camera perspective was required. Thus, despite some compromises with respect to the achievable measurement accuracy and precision, an MCV-based approach can be judged to be valid and feasible and, therefore, may revolutionize the collection of kinematic data under in-field conditions in the near future. This might be particularly the case in applications for which large datasets for algorithm training are available.

### 4.4. Methodological Considerations

This study successfully applied and enhanced an existing MCV-based approach for the collection of kinematic data on a ski track. It can be considered an explorative study assessing the potential of such an approach to bring substantial methodological benefit towards the field of sports science and sports medicine. Similar studies on the accuracy of monocular 3D pose estimation have been conducted in lab conditions, for instance, for analyzing forces and load during weightlifting [33]. However, when interpreting the current findings, one should have the following study/method limitations in mind.

First, even though the used reference method (video-based stereophotogrammetry) can be considered the golden standard for an accurate and precise collection of kinematic data on ski track, this method also suffers from systematic and random errors compared to the true 3D human pose. However, a recent study demonstrated these errors to be marginally small; photogrammetric errors were found to be in the magnitude of ~2 cm or lower [2]. Moreover, the in-lab golden standard method of optoelectronic stereophotogrammetry was shown to be seriously constrained for collecting kinematic data on a ski track [1]. Accordingly, the reference method used can be argued to be the best option currently available.

Second, one could argue that compared to other applications of computer vision, the dataset used for the training of the deep nets was rather small. However, in turn, this means that the high accuracy and precision values achieved in the current study are quite remarkable and promising, and that they could have been even better if more training data were available. Augmenting the existing data could be a possible workaround to recording additional datasets. For instance, it was shown that carefully randomized rotation and occlusion of training images leads to improved 2D pose estimation [34].

Third, all reported results are relative metrics, such as positions relative to the hip, joint angles, and lean angles. Estimating absolute position and velocity would need new algorithmic advances that explicitly address the previously mentioned scale ambiguity. The tested method followed Rhodin et al. [21], who centered and scale normalized (crops) the input images before neural network processing. In the current study this process was done manually, but it could be automated using detection methods.

Finally, it is worth mentioning that the transferability of the algorithms performance to other skiing situations, such as from giant slalom to downhill is (due to the specificity of the underlying movement patterns) somehow limited, and that for such alternative purposes the underlying deep nets should always undergo an application-specific training first.

## 5. Conclusions

In summary, this study illustrated the great potential that MVC-based approaches may have, when being applied to characteristic sports settings. For some of the most complex 3D motion patterns in sports (e.g., alpine skiing techniques) and under the most challenging outdoor conditions (on ski track), an accurate and precise 3D human pose estimation has been demonstrated to be both valid and feasible. Moreover, also for skiing-specific metrics, the corresponding accuracy and precision magnitudes were found to be acceptable for detecting relevant differences.

Already after a brief training of the underlying deep nets on an existing dataset, our algorithm performed comparably or slightly better than previously suggested wearable sensor-based measurement approaches. Thus, thanks to an automatization of the annotation process neither requiring complex camera calibration nor manual annotation, and the use of single camera only, for many sportive applications the proposed method may serve as a valuable alternative to existing systems. This is particularly true in cases where high accuracy and precision values within relatively small capture volumes are the aim. However, for larger capture volumes and real-life settings for which no kinematic training data exists, wearable measurement technologies, such as inertial measurements units (IMU) and differential global navigation satellite systems (dGNSS), conventional video-based stereophotogrammetry may still remain the preferred choice.

## Figures and Tables

**Table 1:** Design of the algorithm training, validation, and testing; we followed a six-fold cross validation scheme to report results for all subjects while training on independent parts of the dataset.

	# Training Samples (All Cameras)	S1	S2	S3	S4	S5	S6
**Split 1**	14,941	**Test**	Training	Training	Training	Training	Validation
**Split 2**	14,910	Validation	**Test**	Training	Training	Training	Training
**Split 3**	15,095	Training	Validation	**Test**	Training	Training	Training
**Split 4**	15,192	Training	Training	Validation	**Test**	Training	Training
**Split 5**	15,246	Training	Training	Training	Validation	**Test**	Training
**Split 6**	15,180	Training	Training	Training	Training	Validation	**Test**
**# Validation Samples** **(entire capture volume, all cameras)**	-	3898	3833	3713	3736	3659	3802
**# Test Samples** **(one turn cycle, two cameras)**	-	664	660	640	616	598	626
**# Trials**	-	4	4	4	4	4	4

S1–6: subjects 1 to 6; #: number of.

**Table 2:** Normalized mean per joint position error (NMPJPE) for a monocular computer vision-based 3D reconstruction using different camera views.

	NMPJPE CAM 1 (m)	NMPJPE CAM 2 (m)	NMPJPE CAM 3 (m)	NMPJPE CAM 4 (m)	NMPJPE CAM 5 (m)	NMPJPE CAM 6 (m)	NMPJPE CAM 1–6 (m)
**Test S1**	0.09	0.07	0.07	0.08	0.07	0.08	0.08
**Test S2**	0.08	0.08	0.07	0.08	0.08	0.08	0.08
**Test S3**	0.13	0.08	0.08	0.12	0.07	0.08	0.09
**Test S4**	0.10	0.08	0.07	0.08	0.07	0.08	0.08
**Test S5**	0.09	0.07	0.08	0.07	0.08	0.08	0.08
**Test S6**	0.10	0.07	0.06	0.07	0.07	0.07	0.07
**Mean**	0.10	0.08	0.07	0.08	0.07	0.08	0.08
**SD**	0.02	0.00	0.01	0.02	0.00	0.01	0.01

S1–6: subjects 1 to 6; CAM 1–6: camera 1–6.

**Table 3:** Accuracy and precision of knee and hip flexion angles of the turn outside and inside leg based on a monocular 3D reconstruction (averages over the cameras CAM 3 and CAM 5).

	Test Set	*θ_Knee,outside_* (°)	*θ_Knee,inside_* (°)	*θ_Hip, outside_* (°)	*θ_Hip,inside_* (°)
**Mean error**	Test S1	8.5 ± 7.3	5.5 ± 5.7	4.3 ± 6.7	0.9 ± 4.4
	Test S2	6.1 ± 9.4	2.0 ± 5.2	−2.4 ± 7.4	−1.6 ± 4.8
	Test S3	−8.2 ± 6.0	−0.9 ± 5.5	−8.2 ± 5.0	−5.6 ± 4.7
	Test S4	−9.4 ± 7.0	−13.4 ± 5.5	−0.2 ± 5.7	−3.2 ± 5.0
	Test S5	3.2 ± 6.9	3.0 ± 4.4	1.6 ± 6.7	1.9 ± 4.8
	Test S6	2.3 ± 6.1	2.8 ± 3.8	2.6 ± 5.3	3.6 ± 4.4
**Accuracy**	All subjects	0.4 ± 7.1	−0.2 ± 5.0	−0.4 ± 6.1	−0.7 ± 4.7
**Precision**	All subjects	7.2 ± 1.5	6.7 ± 1.1	4.4 ± 1.5	3.7 ± 1.0

S1–6: subjects 1 to 6. *θ_Knee,outside_*: outside knee flexion angle; *θ_Knee,inside_*: inside knee flexion angle; *θ_Hip, outside_:* outside hip flexion angle; *θ_Hip,inside_*: inside hip flexion angle.

**Table 4:** The accuracy and precision of skiing-specific metrics based on the monocular 3D reconstruction (averages over the cameras CAM 3 and CAM 5).

	Test Set	*p_COM,rel_*	*λ_Lean_* (°)	*d_vertical_* (°)	*d_Fore/Aft_* (°)	*CDA_Barelle* (°)
**Mean error**	Test S1	0.03 ± 0.01	2.8 ± 4.7	0.04 ± 0.03	0.00 ± 0.05	0.01 ± 0.01
	Test S2	0.03 ± 0.01	5.5 ± 4.0	0.02 ± 0.03	−0.03 ± 0.05	0.00 ± 0.01
	Test S3	0.03 ± 0.01	−2.0 ± 3.3	0.00 ± 0.03	0.01 ± 0.05	0.00 ± 0.01
	Test S4	0.03 ± 0.01	−2.0 ± 3.3	−0.01 ± 0.03	0.03 ± 0.05	−0.01 ± 0.01
	Test S5	0.02 ± 0.01	−1.7 ± 3.9	0.01 ± 0.04	0.02 ± 0.05	0.01 ± 0.01
	Test S6	0.02 ± 0.01	−3.3 ± 3.3	0.00 ± 0.03	0.03 ± 0.05	0.01 ± 0.01
**Accuracy**	All subjects	0.03 ± 0.01	−0.1 ± 3.8	0.01 ± 0.03	0.01 ± 0.05	0.00 ± 0.01
**Precision**	All subjects	0.01 ± 0.00	3.4 ± 0.9	0.02 ± 0.01	0.03 ± 0.01	0.01 ± 0.00

S1–6: subjects 1 to 6. *p_COM,rel_*: relative center of mass position (*COM*); *λ_Lean_*: lean angle; *d_Fore/Aft_*: fore/aft position; *d_vertical_*: COM to outside ankle joint center distance; *CDA_BARELLE_*: drag area.
